# Alternate recurrent coronary artery spasm and stress cardiomyopathy: a case report

**DOI:** 10.1186/s12872-020-01760-2

**Published:** 2020-11-04

**Authors:** Yunpeng Jin, Qiming Li, Xiaogang Guo

**Affiliations:** 1 Division of Cardiology, The Fourth Affiliated Hospital of Zhejiang University School of Medicine, N1 Shangcheng Road, Yiwu, 322000 Zhejiang People’s Republic of China; 2 Division of Cardiology, The First Affiliated Hospital of Zhejiang University School of Medicine, N79 Qingchun Road, Hangzhou, 310003 Zhejiang People’s Republic of China

**Keywords:** Case report, Coronary artery spasm, Apical ballooning shape, Stress cardiomyopathy, Myocardial infarction with nonobstructive coronary arteries, Takotsubo syndrome

## Abstract

**Background:**

Coronary artery spasm (CAS) and stress cardiomyopathy (SC) have different characteristic clinical manifestations in the case of suspicious myocardial infarction with nonobstructive coronary arteries. Established recurrence rates of both conditions have been reported, however, alternate recurrent CAS and SC in the same individual have not been described.

**Case presentation:**

A 59-year-old man suffered from atypical chest pain in the first episode, acute heart attack in the second and third episodes (totally 3 times over a period of approximately 5 years). During the first episode, he visited our hospital with mild paroxysmal chest pain without obvious inducement for approximately 2 years. He was underdiagnosed at that time without other obvious findings except the poor R wave progression in V1–3 leads revealed in electrocardiogram. At 4 months after the first episode, he suffered from a heart attack (the second episode) and was diagnosed with SC based on the coronary angiography (CAG) and left ventriculography findings of nonobstructive coronary arteries combined with a classic apical ballooning shape. At 31 months after the second episode, he suffered another heart attack (the third episode) and was diagnosed with CAS based on the CAG results of recoverable severe multivessel stenoses. During the episodes, partial reversible nature of apical hypokinesis was observed in echocardiogram. In retrospect, the patient suffered silent CAS in the first episode, SC in the second episode, and severe multivessel CAS in the third episode.

**Conclusion:**

The unusual presentations observed in this case have not been reported. This case suggests that cardiologists should be aware of the possibility of alternate recurrent CAS and SC in the same individual. Provocative tests for spasm and cardiac magnetic resonance imaging might help gain more insights into this issue.

## Background

Coronary artery spasm (CAS) generally causes transient myocardial ischemia with the manifestations of paroxysmal chest pain and recoverable electrocardiogram (ECG) changes; however, it may also cause life-threatening heart events such as acute myocardial infarction (AMI), lethal arrhythmias, and even sudden death despite its transient nature, especially in the case of multivessel involvement [1].

A definite and timely diagnosis of CAS remains extremely challenging because provocation tests are rarely performed due to the concern regarding its safety [2], although such tests for CAS have been confirmed to be safe and have relevant prognostic implications [3]. Patients are diagnosed with CAS generally based on characteristic clinical manifestations, although atypical scenarios appear occasionally.

Stress cardiomyopathy (SC), also known as Takotsubo syndrome, apical ballooning syndrome, or broken heart syndrome, is a benign disorder characterized by a variety of transient abnormalities in the left ventricular wall motion. Despite its increasing awareness, underdiagnosed and misdiagnosed conditions remain common because of its deceptive clinical manifestations and heterogeneity of clinical findings [4].

CAS and SC have different characteristic clinical manifestations in the case of suspicious myocardial infarction with nonobstructive coronary arteries (MINOCA). Established recurrence rates of both CAS and SC have been reported, however, alternate recurrent CAS and SC in the same individual have not been described.

Herein, we describe a case of alternate recurrent CAS and SC in the same individual, which was observed for the first time.

## Case presentation

A 59-year-old man suffered from atypical chest pain in the first episode and acute heart attack in the second and third episodes (totally 3 times over a period of approximately 5 years). He was a businessman of Han nationality. Past, personal, and family history had no distinct findings, except for a history of smoking one pack of cigarettes per day for 20 years. The timeline of the three episodes are shown in Table 1.

**Table 1:** Timeline of the three episodes

Date	Episode	Main symptoms	Main examination		Medication
2016-10-07	First	Mild paroxysmal chest pain without obvious inducement for approximately 2 years	On arrival	ECG revealed poor R wave progression and suspicious ST-segment elevation in V1–3 leads Echocardiogram findings and cTnT and CK-MB levels were normal Coronary computed tomography angiography showed mild coronary atherosclerotic lesions	He refused to take any medicine at that time
2017-02-15	Second	Chest pain aggravated 1 h ago after waking up (without physical or emotional triggers) at about 7:30 a.m	On arrival	ECG revealed ST-segment elevation in V2–6 leads Emergency CAG was performed that showed no significant atherosclerotic lesions Left ventriculography revealed apical hypokinesis with a classic apical ballooning shape Echocardiogram showed severe apical hypokinesis, and LVEF was 52% CK-MB and cTnT levels were elevated	Aspirin, clopidogrel, statins, angiotensin II type 1 receptor blockers, beta-adrenergic blockers, and insulin. (DAPT stopped after 1 year)
			5 days later	ECG showed recovered ST-segment elevation in V2–6 leads, echocardiogram revealed no significant improvement	
			8 months later	Echocardiogram showed moderate apical hypokinesis, and LVEF was 55%	
			26 months later	ECG revealed no ST-segment elevation, echocardiogram showed mild apical hypokinesis, and LVEF was 68.7%	
2019-10-04	Third	Severe chest pain occurred again 8 h ago when he was resting at about 2:39 a.m	On arrival	ECG revealed ST-segment elevation in II, III, avF, and V1–4 leads. Echocardiogram showed mild left ventricular wall motion reduction, severe apical hypokinesis, and LVEF was 55% Emergency CAG revealed severe stenoses in the left anterior descending and posterior left ventricle arteries, which reversed after intracoronary injection of nitroglycerin CK-MB and cTnT levels were normal	Calcium channel blockers, statins, and insulin
			3 days later	ECG revealed recovered ST-segment elevation in II, III, avF, and V1–4 leads. Echocardiogram showed normal left ventricular wall motion and mild apical hypokinesis, and LVEF was 70.8%	He was insisted on medication and regular follow-up after discharge
		No chest pain	Till date		No drug dose adjusted

*ECG* electrocardiogram, *cTnT* cardiac troponin T, *CK-MB* creatine kinase-MB, *CAG* coronary angiography, *LVEF* left ventricular ejection fraction, *DAPT* dual antiplatelet therapy

### The first episode

**Fig. 1:**
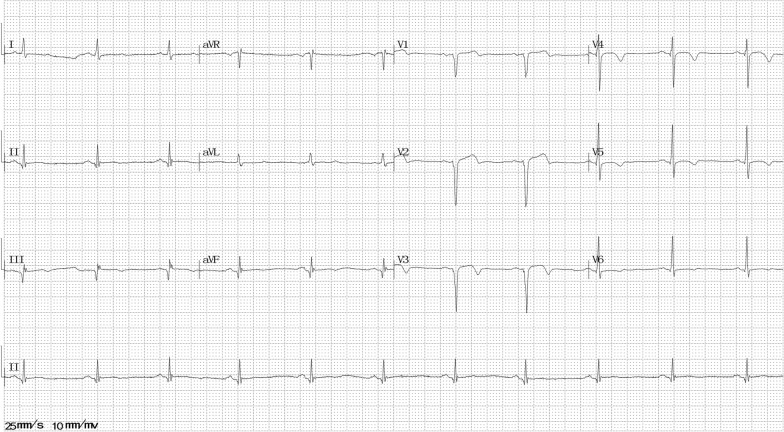
Electrocardiography at the first episode

He visited our hospital with mild paroxysmal chest pain without obvious inducement for approximately 2 years. The chest pain was located in the substernal area, characterized by a squeezing pattern and lasted minutes or hours. ECG revealed poor R wave progression and suspicious ST-segment elevation in V1–3 leads (Fig. 1), whereas echocardiogram findings and cardiac troponin T (cTnT) and creatine kinase-MB (CK-MB) levels were normal. Coronary computed tomography angiography showed mild coronary atherosclerotic lesions. He was diagnosed with diabetes and hypertension simultaneously, but he refused to take any medicine at that time.

### The second episode

**Fig. 2:**
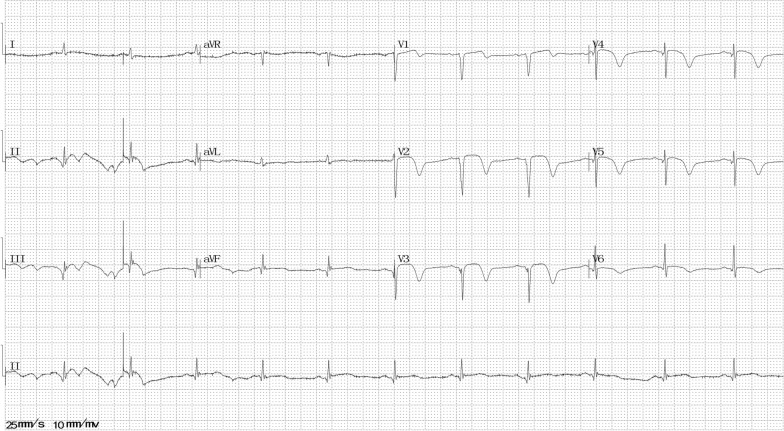
Electrocardiography at the second episode

**Fig. 3:**
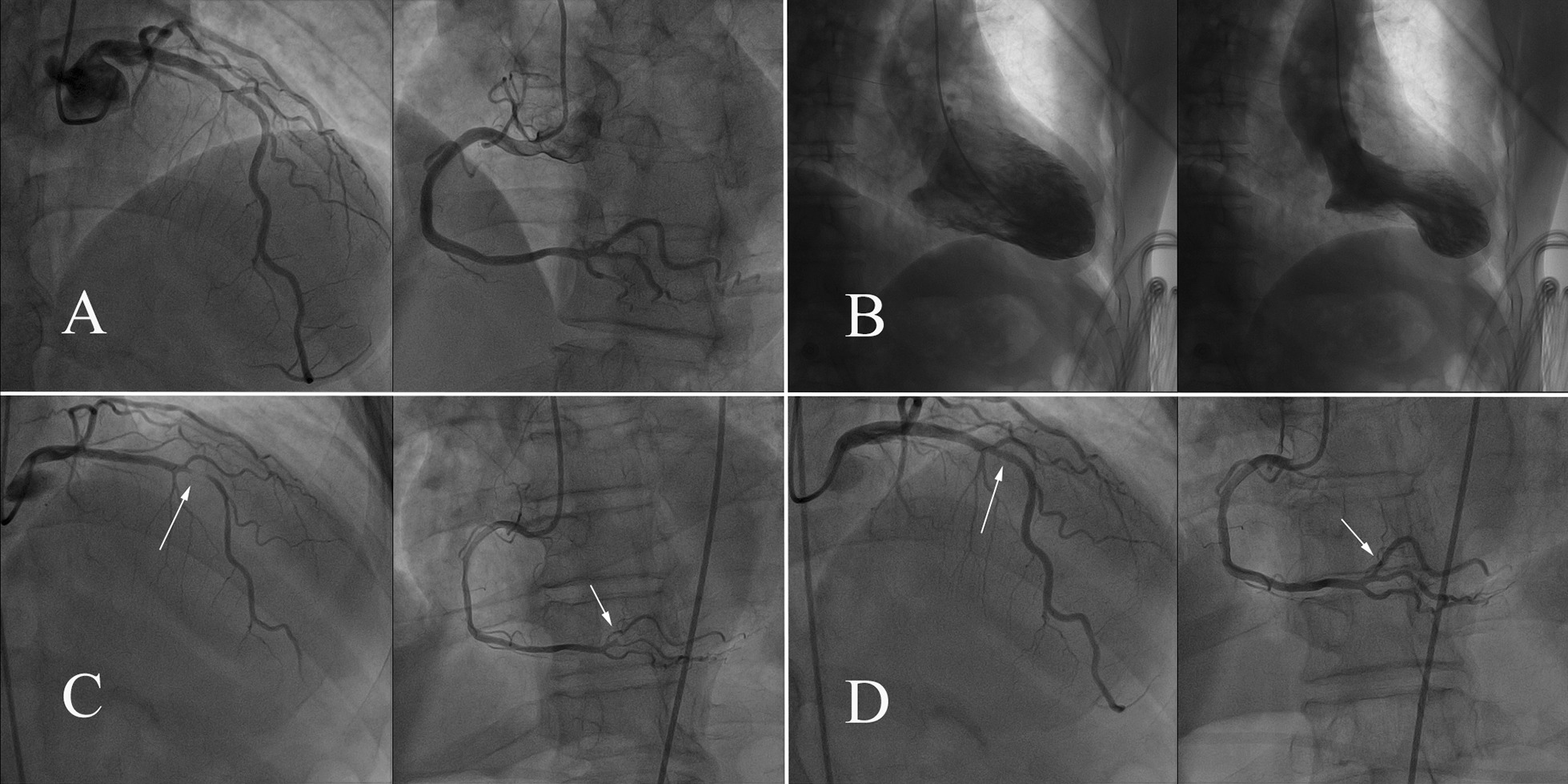
**a** Coronary angiography during the second episode; **b** left ventriculography during the second episode; **c** coronary angiography during the third episode; **d** coronary angiography during the third episode after the intracoronary injection of nitroglycerin

At 4 months after the first episode, he presented to the emergency room with chest pain that aggravated 1 h ago after waking up (without physical or emotional triggers) at about 7:30 a.m. On arrival, his vital signs and physical examination findings were normal. ECG revealed ST-segment elevation in V2–6 leads (Fig. 2). The chest pain was located in the substernal area, characterized by a squeezing pattern and relieved after approximately 30 min before coronary angiography (CAG) was performed. The InterTAK diagnostic score was 18 [5], and the prognostic score was 12 [6]. An emergency CAG was performed that showed no significant atherosclerotic lesions (Fig. 3a). Left ventriculography disclosed apical hypokinesis with a classic apical ballooning shape (Fig. 3b). Additional movie files show this in more detail [see Additional file 1]. Echocardiogram revealed severe apical hypokinesis, and the left ventricular ejection fraction (LVEF) was 52%. Additional image files show this in more detail [see Additional file 2]. Laboratory data showed cTnT levels of 0.021 and 0.796 ng/mL at the peak (relative index < 0.014 ng/mL) and CK-MB levels of 17.2 and 40.4 U/L at the peak (relative index < 24 U/L). His brain natriuretic peptide (BNP) level was 108.1 pg/ml (relative index < 100 pg/ml), low-density lipoprotein cholesterol (LDL-C) level was 1.36 mmol/L (normal range 0–3.36 mmol/L), fasting blood glucose level was 7.34 mmol/L (normal range 3.9–6.1 mmol/L), and glycated hemoglobin A1c (HbA1c) level was 11.2% (normal range 3.6–6.0%). Other examinations showed no remarkable findings. He was diagnosed with SC and treated with long-term medicines, including aspirin, clopidogrel, statins, angiotensin II type 1 receptor blockers, beta-adrenergic blockers, and insulin.

**Fig. 4:**
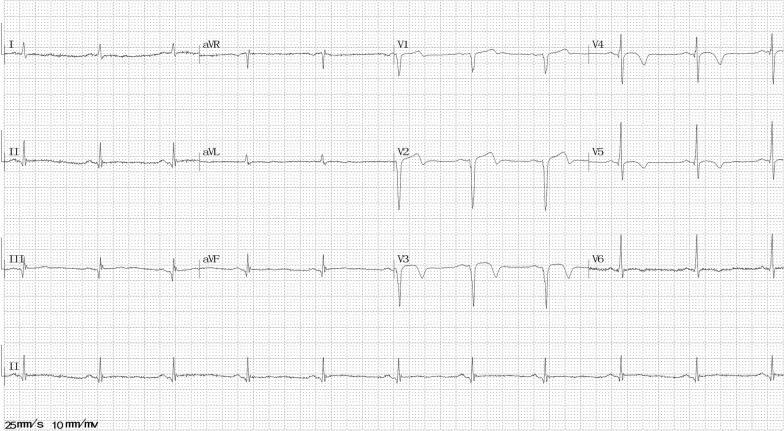
Electrocardiography at 5 days after the second episode

**Fig. 5:**
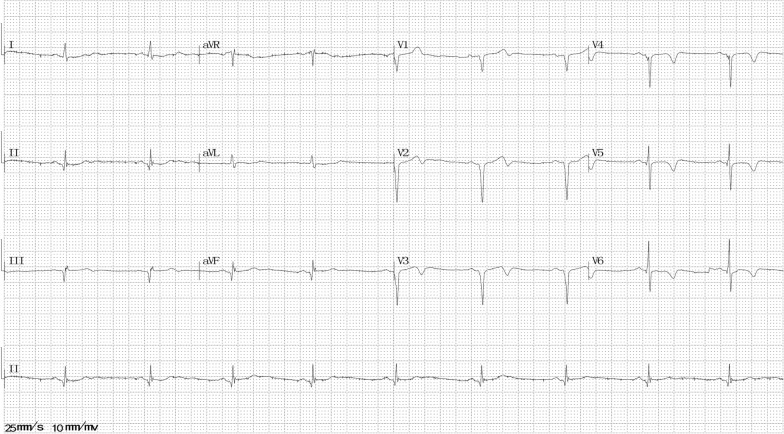
Electrocardiography at 26 months after the second episode

After 5 days, the ECG revealed recovered ST-segment elevation in V2–6 leads (Fig. 4), echocardiogram showed severe apical hypokinesis, and the LVEF was 51%. An additional image file shows this in more detail [see Additional file 3]. After 8 months, the echocardiogram revealed moderate apical hypokinesis, and the LVEF was 55%. After 26 months, no ST-segment elevation was detected on the ECG (Fig. 5), and the echocardiogram revealed mild apical hypokinesis, with the LVEF being 68.7%. Mild paroxysmal chest pain occurred rarely during the follow-up. He did not quit smoking despite our repeated recommendation.

### The third episode

**Fig. 6:**
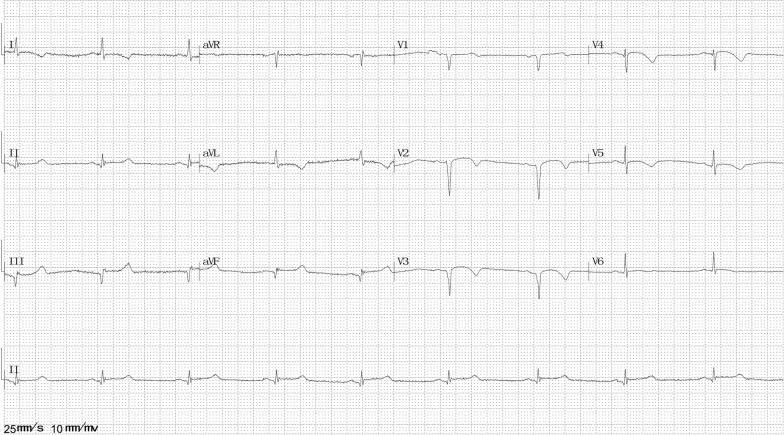
Electrocardiography at the third episode

At 31 months after the second episode, he again visited our emergency room with severe chest pain (squeezing sensation in the substernal area) that occurred 8 h ago when he was resting at about 2:39 a.m. On arrival, his vital signs and physical examination were normal. ECG revealed ST-segment elevation in II, III, avF, and V1–4 leads (Fig. 6). Echocardiogram showed mild left ventricular wall motion reduction, server apical hypokinesis, and the LVEF was 55%. An additional image file shows this in more detail [see Additional file 4]. An emergency CAG revealed severe stenoses (Fig. 3c) in the left anterior descending and posterior left ventricle arteries, which reversed after intracoronary injection of nitroglycerin (Fig. 3d). Additional movie files show this in more detail [see Additional file 5, 6]. Then, the chest pain relieved. His N-terminal pro-BNP level was 266.4 ng/L (relative index < 125 ng/l), fasting blood glucose level was 8.4 mmol/L (normal range 3.9–6.1 mmol/L), and HbA1c level was 10.1% (normal range 3.6–6.0%). Other examinations showed no remarkable findings. He was diagnosed with CAS. An additional treatment with calcium channel blockers (CCBs) was initiated, and beta-adrenergic blockers were stopped.

**Fig. 7:**
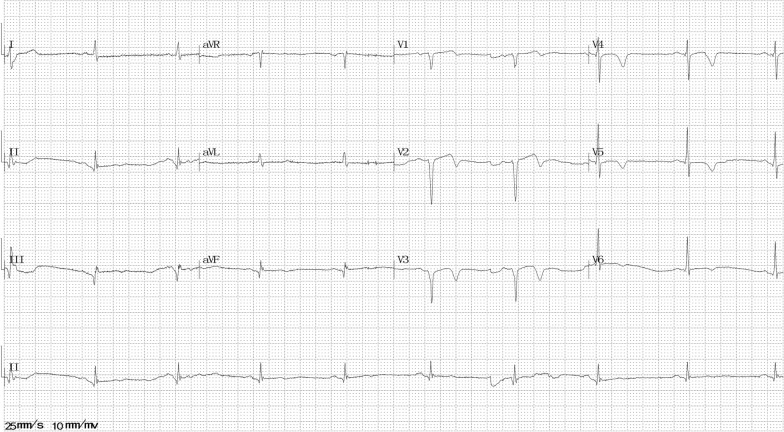
Electrocardiography at 3 days after the third episode

After 3 days, the ECG revealed recovered ST-segment elevation in II, III, avF, and V1–4 leads (Fig. 7), the echocardiogram showed normal left ventricular wall motion and mild apical hypokinesis, and the LVEF was 70.8%. Additional image files show this in more detail [see Additional file 7]. He quit smoking after the second attack. He was insisted on medication and regular follow-up after discharge. No chest pain has occurred till date.

## Discussion and conclusion

### Aspect of diagnosis

This patient presented with suspicious MINOCA in the second episode, and according to the working diagnosis of MINOCA [7], SC and CAS are the most likely diagnosis.

SC was diagnosed initially according to the revised Mayo Clinic criteria at that time, although a definite diagnosis could not be established then because of the uncertain reversible nature of the condition. Partial reversible nature of apical hypokinesis was observed in the echocardiogram during the follow-up. Moreover, the InterTAK diagnostic score is low (only 18 points of 100 points), which have been published as a new recommended diagnostic criteria recently [8]. In other words, the diagnosis of SC during the second episode was still uncertain. However, contrast cardiac magnetic resonance imaging (CMRI) was not performed to detect edema (to confirm SC) at that time due to limited hospital resources.

As multivessel CAS was diagnosed definitely during the third episode, it could have occurred during the second episode as well. Retrospectively, the clinical manifestations are more consistent with the characteristics of CAS than with SC (chest pain occurred at rest in the early morning repeatedly without physical or emotional triggers). Moreover, CAS can diffusely involve the entire arteries and migrate from site to site, which may also cause severe apical hypokinesis with apical ballooning images [9]; multivessel spasm theory [10] also supports this possibility. However, the coronary images showed a wraparound left anterior descending branch, which contradicts this possibility. That it to say, CAS could not be the cause of the second episode. Unfortunately, we did not perform provocative tests, which are known as the key diagnostic criteria of CAS, during the second episode due to the insufficient evidence basis at that time. Recently, provocative tests for spasm have been confirmed to be safe and are recommended in the situation of rest angina without obstructive coronary artery disease [11].

Our understanding of both CAS and SC is still limited. CAS and SC share some predisposing factors and pathogenic mechanisms, such as emotional triggers and adrenergic hyperactivation, and some evidence suggests a potential pathogenic link between these two conditions [12]. In other words, CAS and SC may be two expressions of the same disease, or rather two separate entities with overlapping mechanisms. In the present case, we could not draw the conclusion definitely because of the important limitation that CMRI and provocative tests were not performed.

Another important limitation is that left ventriculography was not performed during the third episode, which could also have been of great help to assess the appearance of a classic apical ballooning shape. However, we believe that both CAS and SC exist in this case, which is because this explanation is the most reasonable for this case as we have discussed earlier.

Furthermore, it is interesting that during the first episode, the patient’s ECG revealed poor R wave progression in V1–3 leads despite normal echocardiogram and CAG findings, which may have resulted due to small areas of prior myocardial infarction with silent manifestation caused by CAS. In addition, the first episode might point to microvascular endothelial dysfunction, and this microvascular dysfunction could be a risk factor for both CAS and SC [12].

In summary, the patient suffered silent CAS in the first episode, SC in the second episode, and severe multivessel CAS in the third episode. Alternate recurrent CAS and SC in the same individual as observed in this case has not been reported till date.

### Aspect of treatment

During the second episode, the patient was discharged on dual antiplatelet therapy (DAPT). However, according to the latest recommendation, in the absence of CAD and in patients with TS or CAS, DAPT increases the risk of bleeding without an obvious benefit in this population, and even the role of aspirin at low doses has discussed over the years [13].

Moreover, beta-adrenergic blockers known as a risk factor for CAS were prescribed according to the treatment principles of SC [14], which may have been the trigger of the third episode. Therefore, beta-adrenergic blockers should be cautiously used before severe multivessel CAS has been completely excluded despite the typical scenarios of SC.

To our best knowledge, the unusual presentations as observed in this case have not been reported in the literature. Cardiologists should be aware of the possibility of alternate recurrent CAS and SC in the same individual. Provocative tests for spasm and cardiac magnetic resonance imaging might help gain more insights into this issue.

## Supplementary information

**Additional file 1.** Left ventriculography during the second episode.

**Additional file 2.** Echocardiogram at the second episode.

**Additional file 3.** Echocardiogram at 5 days after the second episode.

**Additional file 4.** Echocardiogram at the third episode.

**Additional file 5.** Coronary angiography during the third episode.

**Additional file 6.** Coronary angiography during the third episode after the intracoronary injection of nitroglycerin.

**Additional file 7.** Echocardiogram at 3 days after the third episode.

## Data Availability

The datasets used and/or analysed during the current study available from the corresponding author on reasonable request.

## Abbreviations

CAS: Coronary artery spasm
SC: Stress cardiomyopathy
MINOCA: Myocardial infarction with nonobstructive coronary arteries
CAG: Coronary angiography
ECG: Electrocardiogram
AMI: Acute myocardial infarction
cTnT: Cardiac troponin T
CK-MB: Creatine kinase-MB
LVEF: Left ventricular ejection fraction
BNP: Brain natriuretic peptide
HbA1c: Glycated hemoglobin A1c
CCBs: Calcium channel blockers
CMRI: Cardiac magnetic resonance imaging
DAPT: Dual antiplatelet therapy
