# Supplementary figures and images for: Alternate recurrent coronary artery spasm and stress cardiomyopathy: a case report
Source: BMC Cardiovasc Disord. 2020 Nov 4;20:476. doi: 10.1186/s12872-020-01760-2 (PMC7641797; doi:10.1186/s12872-020-01760-2)

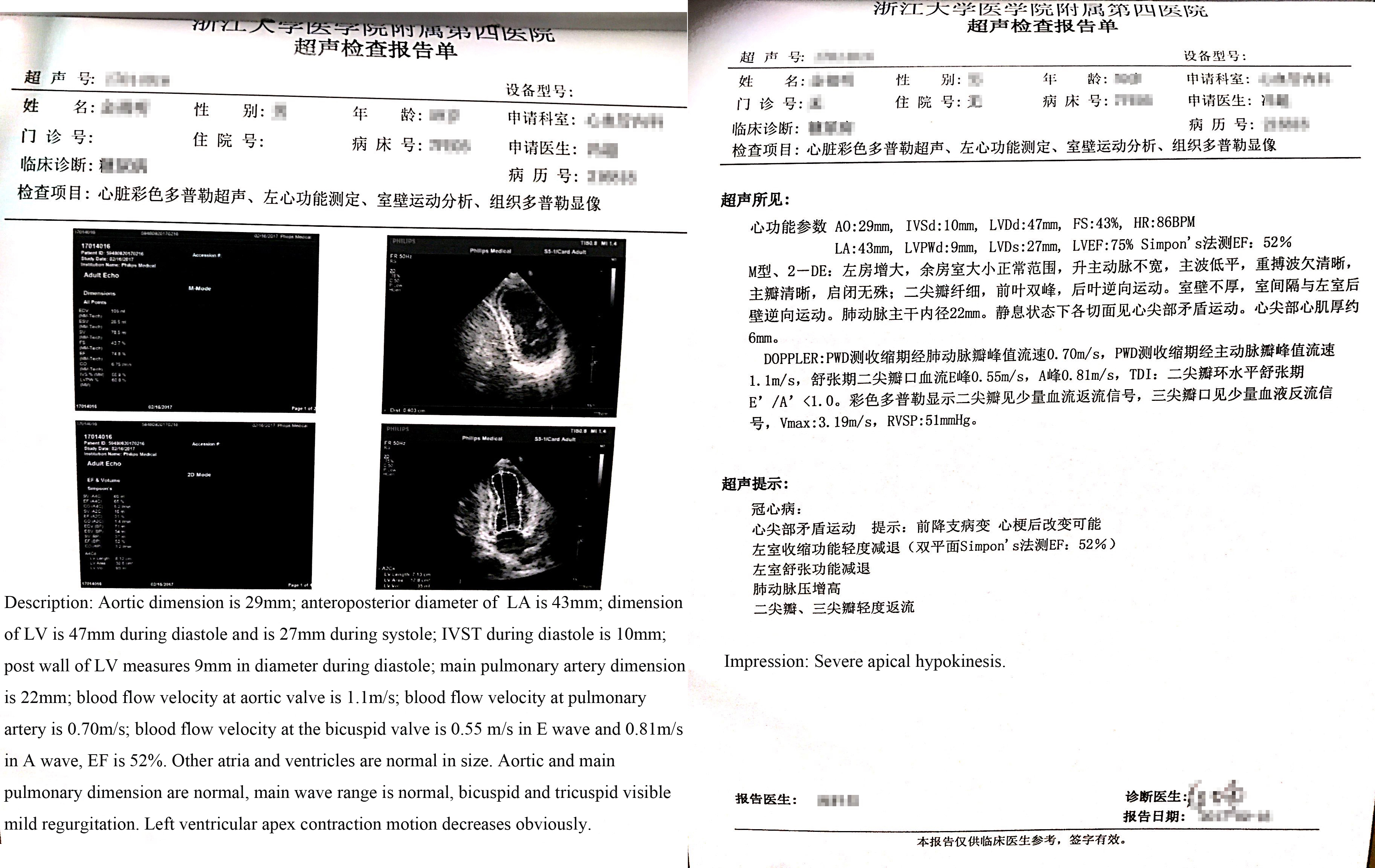

Supplement: Supplementary file 2 — Additional file 2. Echocardiogram at the second episode. [file 12872_2020_1760_MOESM2_ESM.jpg]

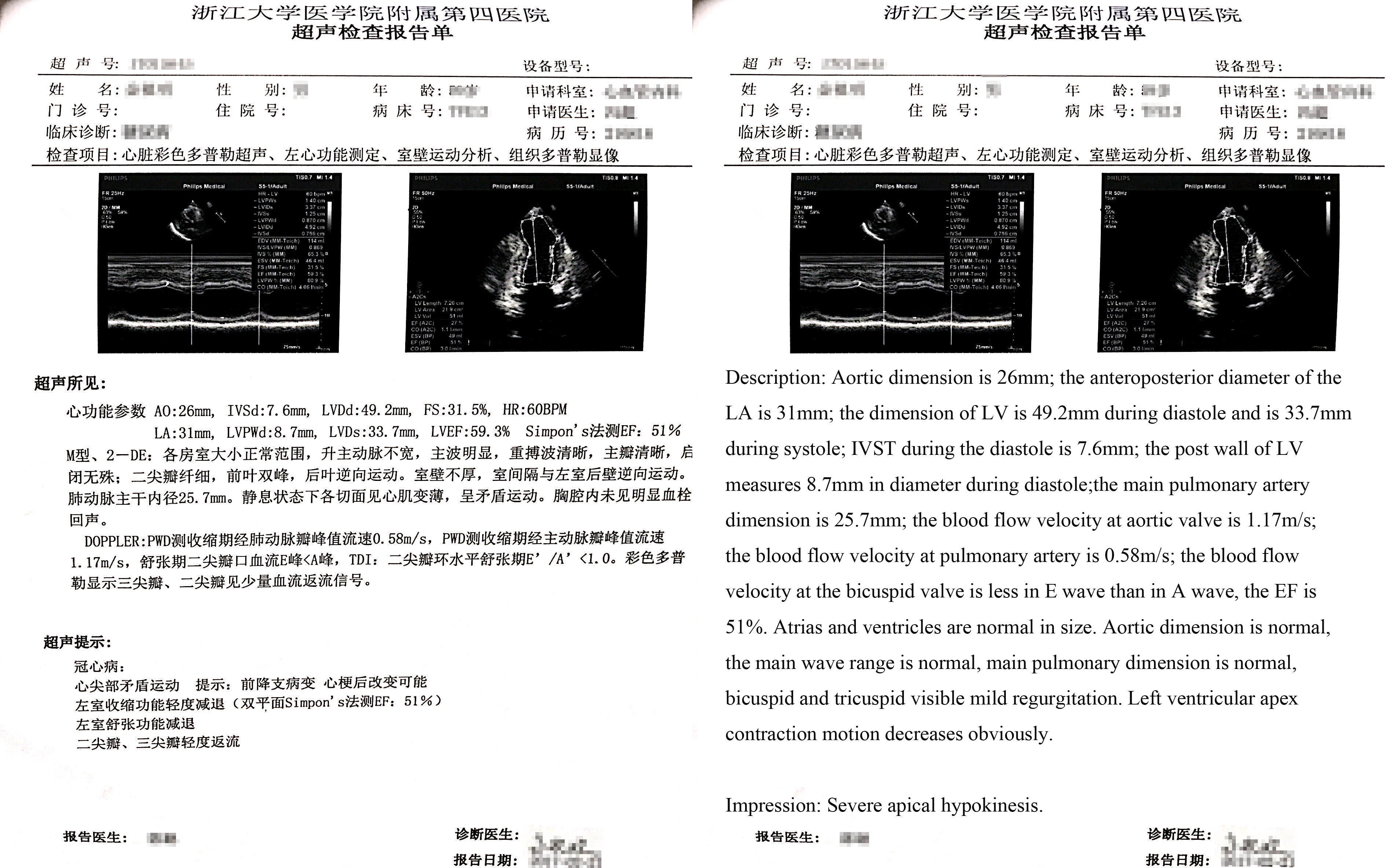

Supplement: Supplementary file 3 — Additional file 3. Echocardiogram at 5 days after the second episode. [file 12872_2020_1760_MOESM3_ESM.jpg]

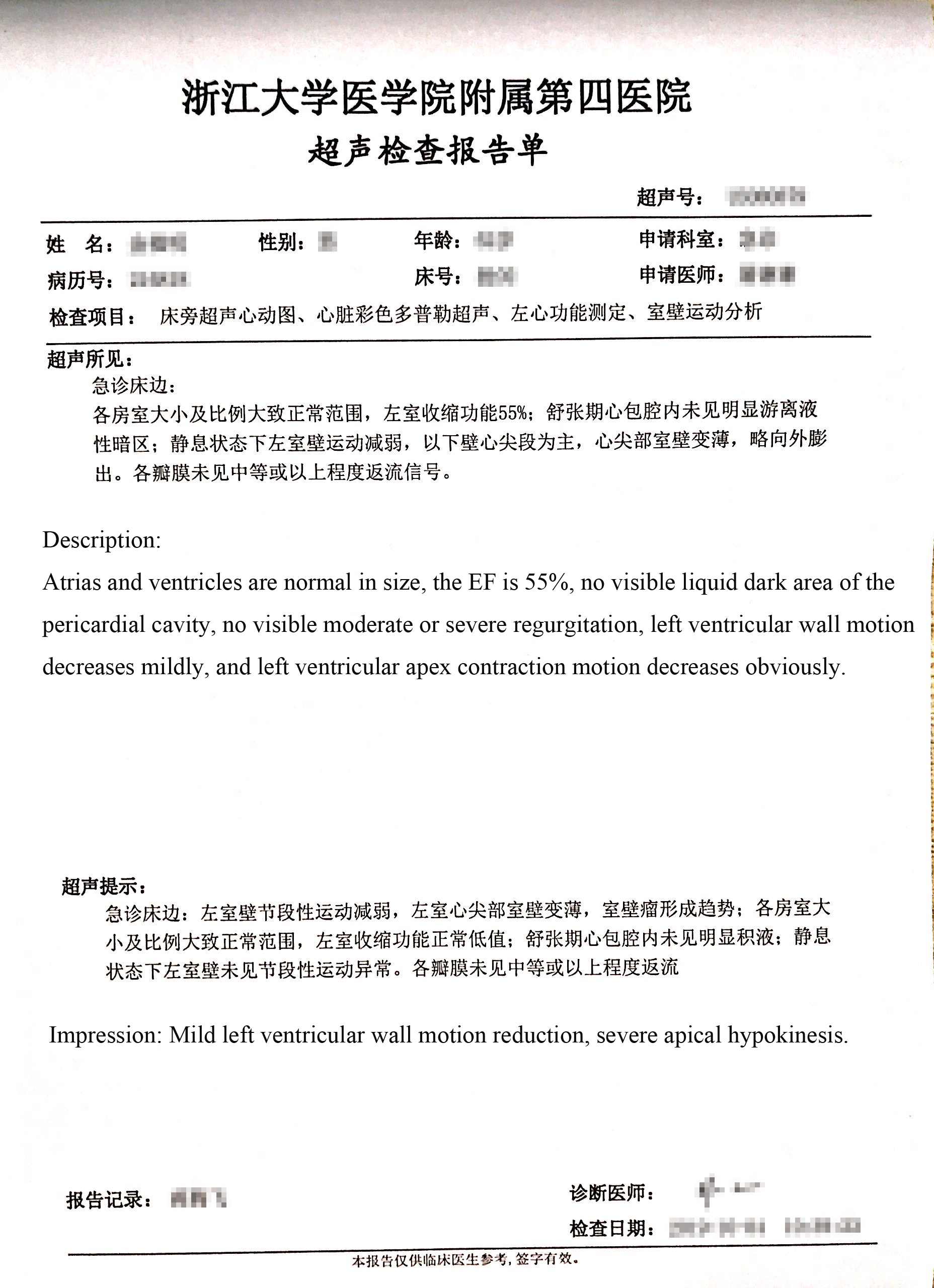

Supplement: Supplementary file 4 — Additional file 4. Echocardiogram at the third episode. [file 12872_2020_1760_MOESM4_ESM.jpg]

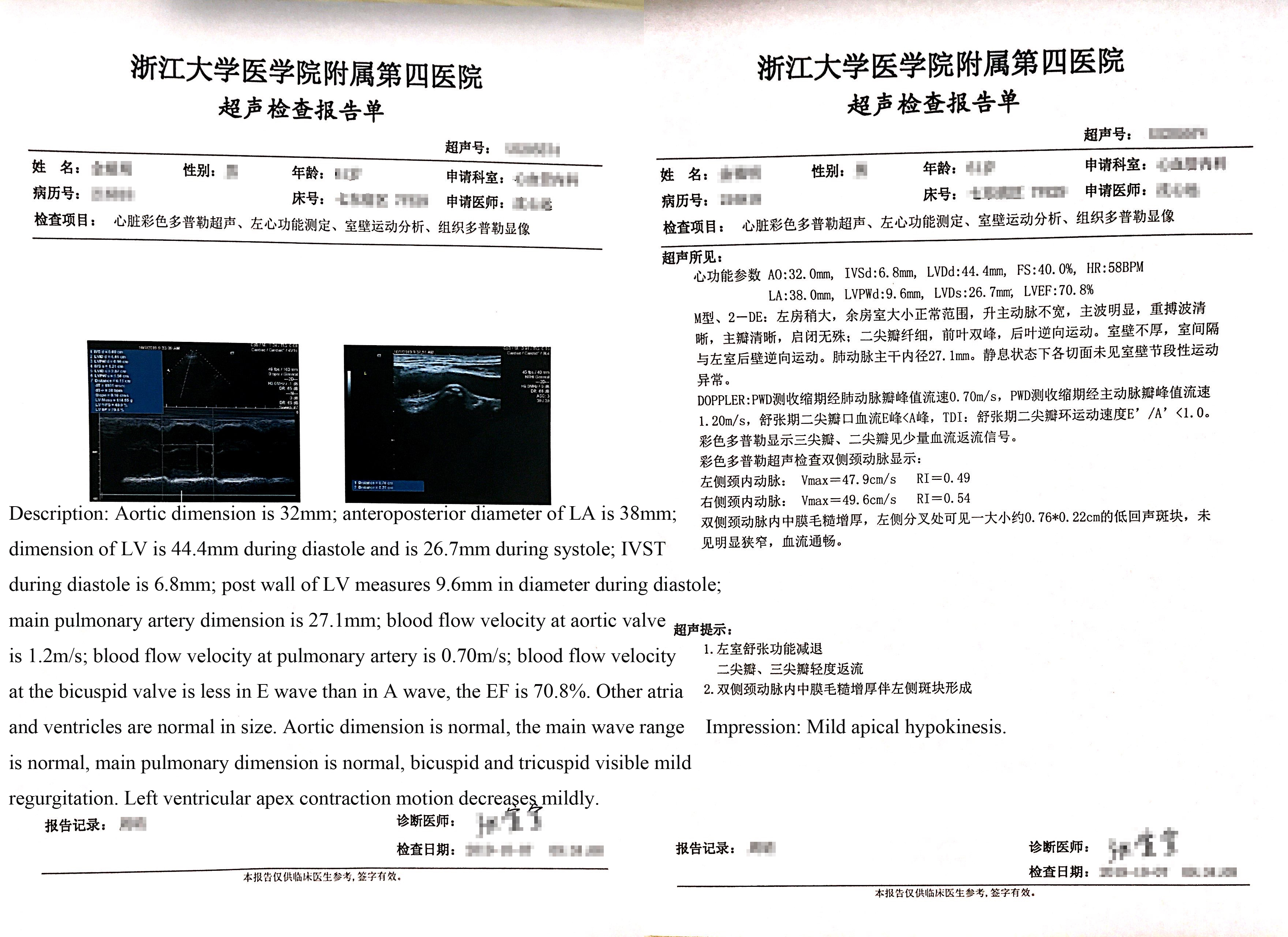

Supplement: Supplementary file 7 — Additional file 7. Echocardiogram at 3 days after the third episode. [file 12872_2020_1760_MOESM7_ESM.jpg]
